# Molecular Insights and Clinical Outcomes of Drugs of Abuse Adulteration: New Trends and New Psychoactive Substances

**DOI:** 10.3390/ijms232314619

**Published:** 2022-11-23

**Authors:** Annagiulia Di Trana, Diletta Berardinelli, Eva Montanari, Paolo Berretta, Giuseppe Basile, Marilyn A. Huestis, Francesco Paolo Busardò

**Affiliations:** 1 Department of Excellence of Biomedical Science and Public Health, University “Politecnica delle Marche” of Ancona, Via Tronto 71, 60124 Ancona, Italy; 2 National Centre on Addiction and Doping, Istituto Superiore di Sanità, 00161 Rome, Italy; 3 IRCCS Orthopedic Institute Galeazzi, 20157 Milan, Italy; 4 Institute of Emerging Health Professions, Thomas Jefferson University, 1020 Walnut St., Philadelphia, PA 19144, USA

**Keywords:** adulterants, drugs of abuse, new psychoactive substances, intoxications, seized drugs, toxicology

## Abstract

Adulteration is a well-known practice of drug manufacturers at different stages of drug production. The intentional addition of active ingredients to adulterate the primary drug may enhance or mask pharmacological effects or may produce more potent drugs to increase the number of available doses and the dealer’s profit. Adulterants found in different drugs change over time in response to different factors. A systematic literature search in PubMed and Scopus databases and official international organizations’ websites according to PRISMA guidelines was performed. A total of 724 studies were initially screened, with 145 articles from PubMed and 462 from Scopus excluded according to the criteria described in the Method Section. The remaining 117 records were further assessed for eligibility to exclude articles without sufficient data. Finally, 79 studies were classified as “non-biological” (*n* = 35) or “biological” (*n* = 35 case reports; *n* = 9 case series) according to the samples investigated. Although the seized samples analyses revealed the presence of well-established adulterants such as levamisole for cocaine or paracetamol/acetaminophen for heroin, the reported data disclosed new adulteration practices, such as the use of NPS as cutting agents for classic drugs of abuse and other NPS. For example, heroin adulterated with synthetic cannabinoids or cocaine adulterated with fentanyl/fentalogues raised particular concern. Notably, adulterants play a role in some adverse effects commonly associated with the primary drug, such as levamisole-adulterated cocaine that may induce vasculitis via an autoimmune process. It is essential to constantly monitor adulterants due to their changing availability that may threaten drug consumers’ health.

## 1. Introduction

In 2020, about 284 million people consumed drugs within the previous year, representing an increase of 26% compared to 2010. Cannabis products remain the most abused compounds all over the world, while the highest prevalence of drug-related deaths is attributed to opioids [1]. Over recent decades, the drug market continuously evolved in response to several factors, including recent laws banning substances and precursors, but also events involving the entire world such as the recent SARS-CoV-2 pandemic [2]. Between 2010 and 2020, an alarming increase in drug seizures was observed in Europe, reflecting a 477% increase in methamphetamine seizures, 278% increase in herbal cannabis seizures, 266% increase in cocaine seizures, and a decrease in heroin seizures [3]. Although seizures data reflect only partially the drug supply chain, it suggests that drug market dynamics are changing.

Besides the threat of illicit drug consumption, adulteration with other active compounds represents an additional risk for drug users, exposing them to unanticipated intake of a wide range of pharmacologically active substances. Adulteration is a well-known practice of drug manufacturers at different stages of drug production [4,5]. Different from drug dilution and drug counterfeiting, the intentional addition of active ingredients to adulterate the primary drug may enhance or mask pharmacological effects or may produce more potent drugs to increase the drug dealer’s profit [6]. Furthermore, most of the adulterants are not internationally banned unless they are controlled under national health or food regulations [7]. Adulterants found in different drugs changed over time in response to different factors, such as controls implementation. In the last decades, the increased controls on methylenedioxymethamphetamine (MDMA) and its synthetic precursors affected the drug manufacturing process leading to poorer quality “ecstasy” tablets with increased adulterants [8]. On the other hand, some cutting agents frequently reoccur in the manufacturing process such as levamisole for cocaine or paracetamol and xylazine for heroin [4,5,7].

Recently, concerns were raised by the adulteration of low ∆-9-tetrahydrocannabinol (THC) cannabis products with more powerful synthetic cannabinoids [9]. Besides synthetic cannabinoids, other new psychoactive substances (NPS)-adulterated classical drugs of abuse have provoked a wave of fatal intoxications around the world. Fentanyl and its analogues were employed for decades as heroin adulterants; but recently “nitazenes”, new synthetic benzimidazole opioids with high potencies, are found as common opioid-cutting agents [10,11].

Here, we review the most recent reports of adulterants detected in seized drugs of abuse including NPS. Furthermore, we reviewed reported cases of adulterated drug intoxications published in the literature from 2017 to 2022, focusing on clinical signs, pharmacological peculiarities, reported intoxication symptoms, and toxicological analyses performed on different human specimens.

## 2. Results

**Figure 1:**
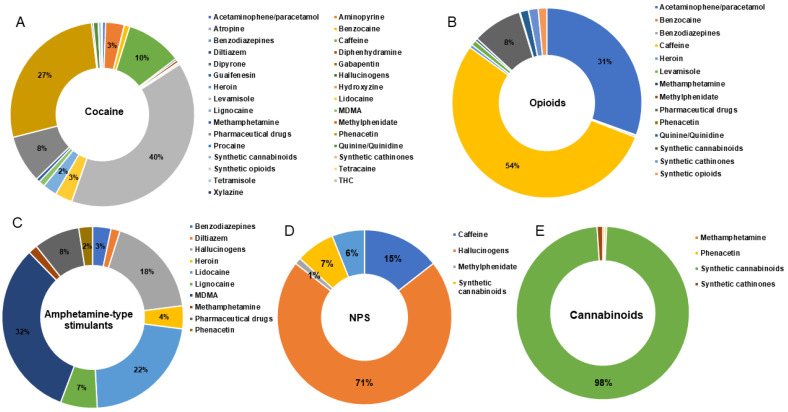
Adulterants prevalence in cocaine (**A**), opioids (**B**), amphetamine-type stimulants (**C**), new psychoactive substances (NPS, **D**) and cannabinoids (**E**) seized samples.

**Figure 2:**
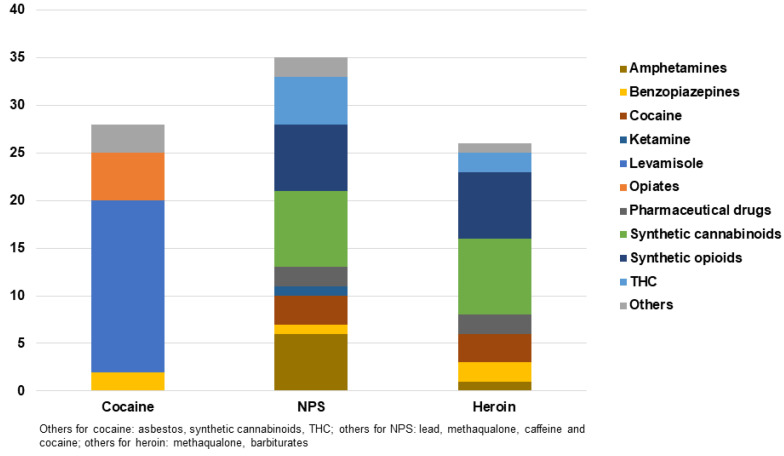
Cocaine, new psychoactive substances (NPS) and heroin adulterants detected in biological samples of intoxication cases reported between 2017 and 2022.

A total of 724 studies were initially screened, with 145 articles from PubMed and 462 from Scopus excluded according to the criteria described in the Method Section. The remaining 117 records were further assessed for eligibility to exclude articles without sufficient data. Finally, 79 studies were classified as “non-biological” (*n* = 35) or “biological” (*n* = 35 case reports; *n* = 9 case series) according to the samples investigated. All results are summarised in Table 1, Table 2 and Table 3 and Figure 1 and Figure 2.

### 2.1. Non-Biological Samples

The literature search yielded 35 studies (Table 1) on seized cocaine (*n* = 20), opioids (*n* = 14), amphetamine-like stimulants (*n* = 8), NPS (*n* = 2), and THC (*n* = 2). Although most manuscripts considered one adulterated drug, different substance classes were investigated in 7 studies [12,13,14,15,16,17]. Cocaine was the most frequently adulterated drug, whereas the highest number of adulterants were detected in amphetamine-like stimulants that were adulterated with 22 different substances [12].

Levamisole and phenacetin were the most prevalent cocaine adulterants, followed by caffeine. The reported adulteration with local anaesthetics such as lidocaine, benzocaine, or procaine [12,13,16,18,19,20,21,22,23,24,25] is a known practice to mimic the highly pure cocaine anaesthetic effect on the mucosa. Although fentanyl traces were detected in cocaine samples seized in the USA [26], other drugs of abuse or pharmaceuticals were rarely reported in cocaine street samples [12,13,16]. The investigated opioids were heroin, fentanyl, morphine, opium, and “crack heroin”. The largest number of adulterants were found in fentanyl samples [27,28], primarily synthetic opioids, synthetic cathinones, caffeine, and acetaminophen. Paracetamol/acetaminophen and caffeine were confirmed as the most common opioids cutting agents according to Tittarelli et al. [5]. Fentanyl/fentalogues adulterated heroin was reported in 3 cases in Canada and in the USA [13,29,30], whereas heroin adulterated fentanyl was detected only in the USA and Canada [27]. Other psychotropic substances such as cocaine, methamphetamine, and benzodiazepines were detected in opioids seized samples. Lead was the most prevalent adulterant in opium samples, but it was detected also in heroin, methamphetamine, and ecstasy seized in Iran [15,31]. It was proposed that lead was added to heroin and other drugs during the first phases of the supply chain, to increase the weight of the batch and obtain more profit.

Amphetamine-type stimulants were adulterated with the most varied compounds, primarily other stimulants, most likely to boost the expected effect. Furthermore, the highest number of NPS (19 different substances in total) were detected in MDMA and methamphetamine seized in the USA and Canada [12,32].

Only 3 studies investigating NPS composition revealed that these adulteration practices also occurred with these compounds that were also used as adulterants of classic drugs [12,31,33] Although data are not sufficient to establish a trend, it seems that NPS are adulterated to enhance some desirable effects with substances producing these effects, such as the synthetic cannabinoids or NBOMe adulterated α-methyltryptamine [33]. 

Cannabis adulteration was the least investigated phenomenon since it was reported in only 2 studies. In this regard, Oomen et al. analysed a large number of samples recently seized in Europe, showing 24% of samples were adulterated with a potent synthetic cannabinoid, MDMB-4en PINACA, [34]. It was apparent that cannabis was adulterated more frequently in e-cigarette cartridges than in flowering tops or edibles.

The quality and quantity of adulterants identified in psychotropic drugs are affected by the analytical approach, as typical targeted methods do not reveal unexpected molecules [23,35]. Examples are on-site drug screening devices that contain antibodies that are specific for a class of drugs and do not react with adulterants. Conversely, the identification and structural characterization of adulterants was primarily conducted via two-step analysis by mass spectrometry and subsequently, nuclear magnetic resonance (NMR) [24,33,36]. Capillary electrophoresis and electrochemical methods are also available [14,23,27,36,37], but the gold standard are hyphenated techniques based on liquid or gas chromatography coupled to mass spectroscopy [12,15,17,20,22,28,29,30,38,39,40,41,42,43,44].

### 2.2. Biological Samples

A total of 35 case reports of drug adulterants’ intoxications are included (Table 2). Adulterants were analytically detected in biological matrices in only 15 cases and identified compounds were levamisole (*n* = 7), 5F-MDMB-PICA (*n* = 1); lead (*n* = 1); sildenafil (*n* = 1); etizolam and caffeine (*n* = 1), and lysergic acid diethylamide (LSD) (*n* = 4) [33,42,45,46,47,48,49,50,51,52]. In the remaining 20 cases, adulterants were only hypothesized based on the observed symptoms, disease or indirect sequelae or confiscated drug analysis. Suspected substances were levamisole (*n* = 10), methaqualone and several benzodiazepines, caffeine and acetaminophen (*n* = 1), 5F-MDMB- PINACA (*n* = 7) and phenethylamine (*n* = 1), asbestos (*n* = 1) [33,42,44,45,46,47,52,53,54,55,56]. Considering all of the reported cases, levamisole was the most prevalent adulterating agent in street cocaine (*n* = 17 cases) [33,42,44,45,46,47,54,57,58,59,60,61,62].

In this regard, the most interesting association between an adulterant and resultant disease occurred with levamisole and perinuclear or cytoplasmic anti-neutrophil cytoplasmic antibodies (p- or c-ANCA) positivity. P- or c-ANCA are two autoantibodies which target different neutrophil proteins: myeloperoxidase (MPO) associated with p-ANCA and proteinase3 (PR3) associated with c-ANCA. Generally, ANCA positivity is a marker of autoimmune pathologies including systemic autoimmune vasculitis, necrotising crescentic glomerulonephritis, inflammatory bowel disease and other autoimmune pathologies [63]. In levamisole-adulterated cocaine intoxications [33,44,45,46,47,54,57,58,59,60,61,62] the p- or c-ANCA positivity was associated with Cocaine Induced Midline Destructive Lesions (CIMDL) (*n* = 5) [44,57], vasculitis (*n* = 8) [33,47,58,59,60,61], membranous glomerulonephritis (*n* = 4) [58,62], pyoderma gangrenosum (*n* = 1) [54], neutropenia complicated with bowel necrosis (*n* = 1) [46], and cerebral white matter dysfunction (*n* = 1) [45]. These autoimmune pathologies were more frequent in women (100% ANCA positivity) than men (90% of ANCA positivity) [64]. Vasculitis was also observed in a p-ANCA-negative cocaine user disregarding the expected and known association between p/c ANCA positivity and vasculitis [33].

The average age of the cocaine users that experienced levamisole toxicity due to adulteration was 44 years, including 11 males (average age: 41.8 years) and 7 females (average age: 45.3 years). Age and sex were factors related to levamisole and cocaine exposure in the different pathologies. CIMDL was associated with younger age and female sex (36.3 years, 60% women), older women experienced more vasculitis (46.5 years, 60% female) and older men more glomerulonephritis (42 years). 

Levamisole was analytically confirmed in only 7 cases, with suspected exposure based on the clinical manifestations and confirmed cocaine exposure [33,44,45,58,59]. Qualitative levamisole analysis was frequently performed in urine (*n* = 5), while the quantitative confirmatory analysis was performed on hair (*n* = 2) with segmental analysis. Levamisole-adulterated cocaine produced toxicity in the nasal septum including CIMDL (2), kidney (2), brain (1) and skin vessels (2). 

Adulterated opioids were found in 10 intoxications. The most frequent adulterated opioid was heroin laced with 5F-MDMB-PINACA (*n* = 8) [52], while methaqualone combined with oxazepam, ketazolam, nordiazepam, pinazepam, and alprazolam was detected in one heroin consumption case [55]. Lead was detected in one opium abuse case characterized by non-specific symptoms including nausea, acute/severe abdominal pain, and vomiting [49]. Patients with severe abdominal pain may undergo unnecessary emergency abdominal surgery, due to a diagnosis of occluded bowel rather than chronic lead intoxication. The average age of individuals experiencing heroin-adulterated intoxication was 30.4 years, with 8 male and two female heroin users reported as exposed to adulterants. Urine was analysed in 10 cases, while serum confirmation was conducted only in 3 cases. Urinalysis revealed the presence of other psychotropic substances combined with heroin including other opioids (*n* = 7), fentanyl (*n* = 5), and cocaine (*n* = 3) [49,52,55]. 

The most frequent substances detected in serum were 6-acetylmorphine (*n* = 6), fentanyl (*n* = 3) and heroin (*n* = 3), while 5F-MDMB- PICA and cocaine were positive for 1 case [52]. Drug analysis clarified the cause of intoxication revealing the presence of 5F-MDMB- PINACA (*n* = 7), fentanyl (*n* = 6) as the most detected analytes. [52] Methaqualone was detected in only one case. [55] While all adulterated heroin cases were associated with other drugs, cocaine was adulterated with levamisole in only 6 out of 17 and was also associated with secondary drugs, such as THC, benzoylecgonine, ecgonine methyl ester, norcocaine and tramadol, cannabinoids, opiates, benzodiazepines, opiates, methadone [45,53,58,59,61]. LSD, γ-hydroxybutyric acid (GHB) and kratom were adulterated in 7 cases, involving only males, with an average age of 30.3 years [48,50,51]. The most investigated biological matrix was urine (*n* = 5), followed by blood (*n* = 4), serum (*n* = 3) and hair (*n* = 1). In all these cases, quantification was accomplished with sophisticated analytical methods. The adulterants were THC and MDMA for LSD (*n* = 3), sildenafil (*n* = 1) for GHB, etizolam (*n* = 1) for alprazolam and phenylethylamine (*n* = 1) for kratom [48,50,51].

### 2.3. Case Series Reports

Among the 9 case series reports, cocaine and heroin were the main adulterated drug identified between 2017 and 2022 (Table 3). 

The most frequent cocaine adulterant was levamisole, detected in hair, urine and postmortem brain [43,65,66,67,68,69].

In 2019, Handley et. al. found higher levamisole concentration in urine than in plasma (565 ng/mL vs 10.6 ng/mL), due to its rapid metabolism (t_1/2_ = 5.6 h). Interestingly, levamisole was more concentrated in brain tissue than in the other matrices (128 ng/mg) suggesting an accumulation in the parenchyma and facilitated passage through the blood–brain barrier [66].

In the series of heroin cases, acetylfentanyl, furanylfentanyl, U-47700, and norfentanyl were the adulterants identified in segmented hair (*n* = 40), urine (*n* = 30), and oral fluid (*n* = 30). Concentrations in hair were 2.3–8600 pg/mg for fentanyl (mean = 860 pg/mg, median = 440) and 2.1–3200 pg/mg for acetylfentanyl [70,71]. Urine and oral fluid analyses were qualitative. Fentanyl and synthetic opioids (acetylfentanyl, furanylfentanyl, norfentanyl and U-47700) were detected in the hair of individuals enrolled in opioid substitution therapy.

**Table 1:** Adulterants detected in seized samples of cocaine, opioids, amphetamine-type stimulants, and new psychoactive substances (NPS) reported in the literature between 2017 and 2022.

	Drug Class #Cases	Adulterant #Cases	Analysis	Seizure Yrs Country	Ref.		Drug Class #Cases	Adulterant # Cases	Analysis	Seizure Yrs Country	Ref.
Cocaine						Opioids					
1	7841	Levamisole; phenacetin; caffeine; lidocaine	GC-MS	2006–2015 Switzerland	[20]	33	Fentanyl 2	U-47700; lidocaine; heroin; 6-AM; methoxyacetyl fentanyl; phenyl fentanyl; codeine; methacryl fentanyl; caffeine	GEMBE	2019 USA	[27]
2	728	Levamisole 433; phenacetin 323; aminopyrine 60; benzocaine 29; lidocaine 22; caffeine 21	FTIR-MCR-ALSGC-FID	2009–2015 Brazil	[21]	34	Fentanyl 46	Xylazine 46	GC-MS LC-HRMS/MS	2020 Australia	[28]
3	24	Tetramisole 23; levamisole 1	LC-MS/MS	2013–2016 Switzerland	[43]	35	Heroin 4	Ocfentanil 4; caffeine 4; paracetamol 4	GC-MS LC-MS/ MS	2015 Spain	[30]
4	351	Levamisole 214; lignocaine 103; meth 13; MDMA 10; phenacetin 7; benzocaine 6; THC 4; amphetamine 1; heroin 2	GC-MS	2015–2016 Canada	[12]	36	Heroin 217; cocaine 19; amphetamine 367	Caffeine 852; nicotine 244; acetaminophen 261; levamisole 8	GC-MS	2015–2016 Hungary	[44]
5	292	Levamisole 292	SVM-DA GC-FID	2015 Belgium	[37]	37	Heroin 11	Meth 7; dextromethorphan 3; cocaine 2; diazepam 1	GC-MS	2015–2016 Canada	[12]
6	97	Levamisole 92; phenacetin 22; caffeine 14; hydroxyzine 10; benzocaine 6	GC-MS GC-TMS UPLC-MS/MS	2016–2017 USA	[17]	38	Heroin 10	Lead 10; caffeine 8; chloroquine 3; methadone 3; phenobarbital 2; tramadol 2; acetaminophen 1; meth 2	HPLC-MS GC-MS FAAS	2016–2017 Iran	[15]
7	17	Caffeine 11; lidocaine 10; acetaminophen 9; phenacetin 7; diltiazem 4	HPLC-DAD	2017 Brazil	[22]	39	Heroin 198	Quinine/quinidine 44; fentanyl 31; lidocaine 24; caffeine 21; levamisole 20; phenacetin 12	GC-MS LC-Q-ToF	2017 USA	[13]
8	96	Levamisole 30; lidocaine 26; phenacetin 13; caffeine 11; quinine/quinidine 12	GC-MS LC-Q-ToF	2017 USA	[13]	40	Heroin 7	Acetaminophen 4; caffeine 4; fentanyl 1	GC-MS	2018 UK	[29]
9	3	Caffeine 3	Colorimetric GC-FID	2018 Brazil	[21]	41	Heroin 30	Caffeine 30; noscapine 26; papaverine 22; paracetamol 16; lidocaine 2; dextromethorphan 5	GC-MS GC-FID SWV	2018 Belgium	[72]
10	50	Levamisole 17; lidocaine 16	CE-C^4^D	2018 Brazil	[23]	42	Heroin 3	Caffeine 1	GC-MS GC-VUV	2019 USA	[38]
11	3	Levamisole 3	SWV HPLC-MS GC-MS GC-FID NMR	2018 Belgium	[36]	43	Heroin 59 Meth 26 Oxycodone	Caffeine 7	GC-MS	2019 Australia	[39]
12	3	Levamisole 3	CV	2018 Belgium	[14]	44	Heroin 659	Paracetamol 656; caffeine 656	GC-MS HPLC-UV LC-Q-ToF	2019–2020 Luxembourg	[16]
13	7	Levamisole 5; Phenacetin 3; caffeine 2; procaine 1; tetracaine 1	GC-MS NMR	2018 France	[24]	45	Heroin 1	4-AP-t-BOC (tert-butyl-4-anilinopiperidine-1-carboxylate)	GC-MS LC-MS	2019–2020 Ireland	[40]
14	156	Levamisole 58; caffeine 39; quinine/quinidine 27; diphenhydramine 20; phenacetin 17; acetaminophen 17; lidocaine 16; procaine 13; diltiazem 10; xylazine 4; atropine 2; aminopyrine 4; hydroxyzine 3; dipyrone 2; guaifenesin 1; gabapentin 1	GC-MS LC-Q-ToF	2018 USA	[25]	46	Opium 10	Lead 10; acetaminophen 8; chloroquine 4; tramadol 2	HPLC-MS GC-MS FAAS	2016–2017 Iran	[15]
15	47	Levamisole; phenacetin; lidocaine; caffeine	DART-HRMS	2019 China	[18]	47	Opium 10	Lead 10	AAS	2015–2016 Iran	[31]
16	1078	Levamisole 584; phenacetin 386; caffeine 113; lidocaine; ketamine; hydroxyzine	GC-MS HPLC-UVLC-Q-ToF	2019–2020 Luxembourg	[16]	48	Morphine 6	Codeine 4; thebaine 2; papaverine 2; noscapine 2; 6-AM 1; heroin 1; oxycodone 1; acetylcodeine 1; fentanyl 1; alprazolam 1	GC-MS	2015–2016 Canada	[12]
17	169	Phenacetin 169	GC-EA-IRMS	6 yrs France	[41]	49	Fentanyl 100	Caffeine 96; etizolam 50; carfentanil 8; cocaine 7; heroin 6; acetyl fentanyl 1	IR SERS PS-MS	2018–2022 Canada	[73]
18	306	Phenacetin 231; caffeine 196; aminopyrine 82; levamisole 16; lidocaine 5; benzocaine 1	GC-MS-QP HPLC-DAD	2019 Uruguay	[19]	**NPS**					
19	45	Caffeine 32; phenacetin 30; levamisole 27; lidocaine 15	GC-MS	2020 Colombia	[42]	50	3-MEC	3-MeO-PCP; 4F-α-PVP; DiPT; ketamine	LC-Q-ToF NMR	2010–2014 Sweden	[33]
	**Amphetamines** **stimulants**					51	4-Aco-DMT	DPT	LC-Q-ToF NMR	2010–2014 Sweden	[33]
20	Amphetamine 28	Meth 25; DMA 14; MDMA 2; cocaine 1; methylphenidate 1; lignocaine 1	GC-MS	2015–2016 Canada	[12]	52	4-Meo-PCP	3-MeO-PCP	LC-Q-ToF NMR	2010–2014 Sweden	[33]
21	Ecstasy 10	Lead 10; meth 4; heroin 2	HPLC-MSGC-MS FAAS	2016–2017 Iran	[15]	52	4-MeO-PCP 2	AB-FUBINACA 2; nicotine 2; ketamine 2; amitriptyline 2	LC-Q-ToF NMR	2010–2014 Sweden	[33]
22	MDMA 211	Unspecified 104; methylone 35; synthetic cathinones 21; methamphetamine 13; benzylpiperazine 8; dextromethorphan 8; mephedrone 5; Amphetamine 4 butylone 4; cocaine 4; ketamine 1; MeO-amphetamine 3; LSD 1	Colorimetric	2010–2015 USA	[32]	53	4-OH-MET	Methoxetamine	LC-Q-ToF NMR	2010–2014 Sweden	[33]
23	MDMA 122	Ethylone 189; meth 34; MDA 16; cocaine 10; 5-Meo-DALT 8; phenethylamine 7; 2C-T-2 6; DMA 5; dibutylone 3; DMT 2; amphetamine 2; methylone 2 sildenafil 2; THC 1 methoxetamine 1; 2C-B 1; MDDM 1;	GC-MS	2015–2016 Canada	[12]	54	α-MT	MDPBP; 25B-NBOME	LC-Q-ToF NMR	2010–2014 Sweden	[33]
24	MDMA 6	Caffeine; lidocaine	GC-MS Raman Spectroscopy	2020 Brazil	[74]	55	Butyrylfentanyl 2	Fentanyl 2; acetylfentanyl 2; 4-ANPP 2	LC-Q-ToF NMR	2010–2014 Sweden	[33]
25	MDMA 302	Unknown 259; dimethylsulfone 36; dimethylsulfone and Caffeine 2; ketamine 2; MDEA 1; cocaine and ketamine 1; N-Moc-MDMA and T-Boc-MDMA 1	GC-MS/MS	2019–2020 Australia		56	Ethylphenidate	5-Meo-DALT; caffeine; phenacetin; lidocaine	LC-Q-ToF NMR	2010–2014 Sweden	[33]
26	Methamphetamine 181	N-isopropyl benzylamine 37; DMA 30; amphetamine 24; cocaine 15; α-PHP 1; DMMA 1; N-Me-2AI 1; ethylphenidate 1; phenethylamine 1; praziquantel 1; 25C-NBOME 1; 25I-NBOME 1; ketamine 1; methoxetamine 1; MDMA 36; THC 36; heroin 3; diazepam 3; alprazolam 1; quetiapine 5; lignocaine 7	GC-MS	2015–2016 Canada	[12]	57	Isopropylphenidate	Methylphenidate	LC-Q-ToF NMR	2010–2014 Sweden	[33]
27	Methamphetamine 10	Lead 10; phenmetrazin e 6; pseudoephedrine 5; caffeine 3; dextromethorphan 3; ketamine 1; MDMA 1	HPLC-MSGC-MS FAAS	2016–2017 Iran	[15]	58	MDMB-CHMICA	AMB-FUBINACA; 1-(cyclohexylmethyl)-1H-indole-3-carbonyl)valine	LC-Q-ToF NMR	2010–2014 Sweden	[33]
28	Methamphetamine 219	Phenacetin 3; caffeine 2; diltiazem 2	GC-MS LC-Q-ToF	2017 USA	[13]	59	MET	3-MeO-PCP/ 4-MeO-PCP; 4-AcO-DMT; 3-MEC; 4F-α-PVP; Ketamine	LC-Q-ToF NMR	2010–2014 Sweden	[33]
**Cannabinoids**						60	Mitragynine	Caffeine	LC-MS/ MS	2010–2014 Sweden	[33]
29	∆9-THC 9	Cocaine 4; 4-F-AMB 3; MDMA 1; methamphetamine 1; 5-UR-144 1; 5F-AKB48 1; desvenlafaxine 1	GC-MS	2015–2016 Canada	[12]	61	NM2201 and THJ-018	MDMB-CHMICA	LC-Q-ToF NMR	2010–2014 Sweden	[33]
30	∆9-THC 1142	MDMB-4en-PINACA 270	GC-MS HPLC-MS UPLC-QToF TLC UPLC-MS3 UPLC-DAD FTIR MALDI-HRMS	2020–2021 Europe		62	THJ-018	MDMB-BB-22	LC-Q-ToF NMR	2010–2014 Sweden	[33]
**Opioids**						63	2C-B 46	5-MeO-DALT 46; MDMA 1	GC-MS	2015–2016 Canada	[12]
31	Crack heroin 10	Lead 10	AAS	2015–2016 Iran	[31]	64	NEP 10	Caffeine 10	GC-MS Raman spectroscopy	2020 Brazil	[31]
32	Fentanyl and analogs 26 Heroin 5	Caffeine 33; AMB-FUBINACA 29; heroin 17; phencetin 10; etizolam 6 AB-FUBINACA 5; fentanyl analogs 6 benzocaine 3; 5F-MDMB-PINACA 3 metformin 2; meth 3 lidocaine 1; acetaminophen 3; dextromethorphan 2; 4Cl-α-PVP 1; UF-17 1; furanyl 5F-MDMB-PICA 1; hydromorphone 1; flubromazolam 1	FTIR Fentanyl immunoassay GC-MS LC-HRMS/MS LC-MS	2018–2019 Canada	[42]						

Abbreviations: 3-MEC, 3-methylethcathinone; 3-Meo-PCP, 3-methoxyphencyclidine; 4-Aco-DMT, 4-acetoxy dimethyl tryptamine; 4-ANPP, 4-anilino-N-phenylpiperidine; 4Cl-α-PVP, 4-Cl-alpha-pyrrolydinovalerophenone; 4F-α-PVP, 4F-alpha-pyrrolydinovalerophenone; 4-Meo-PCP, 4-methoxyphencyclidine; 4-OH-MET, 4 OH methyltryptamine; 5-MeO-DALT, 5-methoxy diallyltryptamine; 6-AM, 6-acetyl morphine; AAS, atomic absorption spectrophotometry; α-MT, α methyltryptamine; CE-C4D, capillary electrophoresis capacitively coupled contactless conductivity detection; CV, cyclic voltammetry; DiPT, diisopropyltryptamine; DMA, dimethylamphetamine; DMMA, dimethylmethamphetamine; DPT, dipropyltryptamine; FAAS, flame atomic absorption spectrometry; FTIR, Fourier transform infrared spectroscopy; GC-VUV, gas chromatography–vacuum ultraviolet spectrophotometry; GEMBE, gradient elution moving boundary electrophoresis; LSD, lysergic acid dimethylamide; MET, methylethyltryptamine; meth, methamphetamine; MDA, 3,4-methylendioxyamphetamine; MDEA, 3,4-methylendioxyethylamphetamine; MDMA, 3,4-methylendioxymethamphetamine; MDPBP, 3,4-methylendioxy-pyrrolidinobutiophenone; NEP, N-ethylpentylone; SERS, surface-enhanced Raman spectroscopy; SWV, square wave voltammetry; TLC, thin layer chromatography.

**Table 2:** Clinical symptoms of authentic cases of adulterated cocaine, heroin and new psychoactive substances (NPS) intoxications reported in the literature between 2017 and 2022.

Clinical Description	Adulterant	Adulterant Analysis	Matrix	Drug Analysis ng/mL	Ref.
Cocaine					
Hx: 52 y F chronic cocaine user admitted with ulcers on extremities; pustule, rash on arms/axilla/temple; longstanding perforation hard palate Lab: c-ANCA+; LA+ Skin biopsy: superficial neutrophil-rich dermal infiltrate, papillary derma edema Dx: pyoderma gangrenosum associated with LAC Tx: prednisone, topical betamethasone	Levamisole *	NA	U	Cocaine +	[54]
Hx: 31 y F cocaine user admitted with facial pain, chronic fronto-maxillary sinus pain, yellow/green discharge, rhinorrhea, crusting, rash legs Lab: ESR 2 mm/h; CRP 24 mg/L; WCC 7.7; p-ANCA+; c-ANCA-; anti-PR3+; anti-MPO Nasal biopsy: ulceration, necrosis surrounded macrophages and fibrotic stroma; chronic inflammatory infiltrate, no vasculitis Dx: CIMDLs, systemic levamisole vasculitis Tx: abstinence, mycophenolate, azathioprine, rituximab, surgical debridement	Levamisole *	NA	U	Cocaine +	[44]
Hx: 30 y F cocaine user, chronic sinusitis, left deviated septum, rhinitis, purulent rhinorrhea, macular rash on hands; Lab: ESR 5 mm/h; CPR 30 mg/L; WCC 11.1; p-ANCA+; c-ANCA-; anti-MPO-; anti-PR3- Nasal biopsy: ulceration, acute/chronic inflammation, fibrotic stroma, vessels permeated by inflammatory cells, no fibrinoid necrosis Dx: CIMDLs and systemic levamisole vasculitis Tx: abstinence, drug rehabilitation, surgical debridement	Levamisole *	NA	U	Cocaine +	[44]
Hx: 36 y M cocaine user, severe facial pain, epistaxis, crusting, rhinorrhoea, rash/livedo in thighs; hematuria, proteinuria Lab: ESR 36 mm/h; CPR 44 mg/L; WCC 8.0; p-ANCA+; c-ANCA-; anti-MPO-; anti-PR3-; IgG4 increase Nasal biopsy (*n* = 3): extensive ulceration, fibrin, proliferative fibrosis; acute/chronic inflammatory cells; no vasculitis (1st); geographic necrosis, fibrinoid necrosis foci (2nd); ulcerated mucosa and granulation; no vasculitis (3rd); Dx: CIMDLs and systemic levamisole vasculitis Tx: abstinence, steroids; methotrexate; mycophenolate; rituximab, surgical debridement	Levamisole	NA	U	Cocaine +; levamisole +	[44]
Hx: 48 y F, severe facial pain, epistaxis, septal perforation with fistula from skin to intra-nasal cavity, soft palate ulceration; hematuria Lab: ESR 80 mm/h; CPR 48 mg/L; WCC 7,6; p-ANCA+; c-ANCA-; anti-MPO-; anti-PR3 + Dx: CIMDLs and systemic levamisole vasculitis Tx: abstinence, steroids, methotrexate, surgical debridement	Levamisole	NA	U	Cocaine +; levamisole +	[44]
Hx: 48 y M, previous Chron’s disease and pneumonia, admitted with purpuric rash worsening in four limbsLab: c-ANCA+; ANA+; anti-MPO- Skin biopsy: leukocytoclastic vasculitis and multiple fibrin thrombi; Dx: levamisole adulterated cocaine-associated vasculitis Tx: supportive therapy	Levamisole *	NA	U	Cocaine +	[60]
Hx: 53 y F cannabis/cocaine user, admitted with extensive, painful retiform symmetric purpura patches in limbs, buttocks/abdomen with necrosis evolution Lab: neutropenia, thrombocytopenia, CPR 13 mg/L; LA-; anticardiolipin antibody-; cryoglobulins-; c-ANCA+ Skin biopsy: thrombotic vasculopathy Dx: levamisole-induced vasculitis Tx: corticosteroids, surgical debridement of necrotic tissue	Levamisole *	NA	U	Cocaine +; THC +	[61]
Hx 46 y M, increased cocaine use last 3 months, admitted with weakness/fatigue, blood-tinged sputum, cough Lab (*n* = 2) creatinine 9.61; GFR 7 mL/min; K+ 7 meq/L (1st); AST 196; ALT 235; total bilirubin 13; A-phosphatase 520 (2nd); ANA+; p-ANCA+; c-ANCA+; anti-α-SME+; anti-HCV+; HCV-RNA- CT chest: cavitary lesions Renal biopsy: fibrous glomerular crescents, few cellular crescents (severe kidney damage) Liver biopsy: isolated intrahepatic bile duct with onion skinning and foci injury, canalicular cholestasis, periportal ductal reaction Dx: intrahepatic duct injury associated with LAC-induced glomerulonephritis Tx: dialysis for AKI; ursodeoxycholic/cholestyramine for duct damage	Levamisole *	NA	U	Cocaine +	[62]
Hx: 29 y M, 3 months cocaine abuse, admitted with severe head-pain, incomprehensible vocalization, amnesia, aggressiveness, agitation, sleep inversion, space/time disoriented; MRI brain (*n* = 2): ubiquitous white matter small lesions (1–14 d after adm); additional punctate lesions in corpus callosum, abn meningeal enhancement, contrast agent in CSF (15–31 d after adm); CSF: protein 224 mg/dL; albumin 37.9; RFA: multiple segmental occlusion in central retinal artery Audiogram: bilateral sensorineural hearing loss; Dx: acute SS associated to LAC; Tx: methylprenisolone, mycophenolate (at brain damage relapse)	Levamisole	UPLC-ESI+-MS/MS	H segments (*n* = 6)	Cocaine >5; BE > 5; EME 0.07–0.11; norcocaine 0.58–1; levamisole 0.07–1.14; tramadol 1.38–2.16	[45]
Hx: 33 y M cocaine abuse history, admitted with 4 weeks of nasal obstruction, dysphagia, otalgia, nasal bridge deformity; Endoscopy: nasal corridor necrosis; CT head/chest: nasal cavity extensive bone destruction, multiple small pulmonary nodules; Lab: leukocytosis, high CRP, high ESR; c-ANCA+; Nasal biopsy: no vasculitis signs; Dx: CIMDL; Tx: counseling on cocaine cessation	Levamisole *	NA	U	Cocaine +; metabolites +	[57]
Hx: 59 y M active cocaine use, anamnestic febrile syndrome with neutropenia/agranulocytosis treated and recovered; new ED admission with 4 days abdominal pain/bloody diarrhea probably due to rectal cocaine administration CT abdomen: rectum/sigmoid/descending colon/caecum/appendix with increased bowel wall thickness Flexible sigmoidoscopy: sigmoid colon/rectum edema, severe ulceration; bowel ischemia/necrosis Lab: normal ENA and ANCA+ Dx: neutropenia agranulocytosis induced by levamisole complicated by bowel necrosis Tx: surgery sigmoid colectomy, ileocecectomy with end ileostomy and descending mucus fistula	Levamisole *	NA	U	Cocaine +	[46]
Hx: 53 y M crack cocaine user, anamnestic hypertension/dyslipidemia/chronic pain syndrome, developed 2 wks limb rash/hematuria/creatinine increase (150 µmol/L); ED admission with abdomen/thighs/legs purpura and ear necrosis Lab: urea 24.8 mmol/L; cr 449; PCR 186.8 mg/mmol; Na = 137 mmol/L; K+ = 4.9 mmol/L; ANA -; ENA -; c-ANCA -; p-ANCA +; HCV/HBV/HIV neg; Renal biopsy: active focal crescentic and necrotizing GN, glomerular capillary wall thickening; tubular epithelial injury; no active vasculitis Dx: concurrent AAV secondary to LAC and associated MN Tx: corticosteroids, ACE inhibitor	Levamisole *	CEDIA	U	Cocaine metabolites (s) +	[58]
Hx: 35 y M tobacco/cannabis smoker, intranasal cocaine use, inhaled crack use; admitted to ED with hemoptysis, iron deficiency anemia CT chest: bilateral ground glass opacities; Lab: urea 7.1 mmol/L; Cr 150 µmol/L; Na 136 mmol/L; K+ 4 mmol/L; ANA -; c-ANCA -; p-ANCA +; cryoglobulinus -; HBV/HCV/HIV neg; Renal biopsy: active segmental fibrinoid necrosis, no arteries vasculitis, diffuse epithelial cell foot process effacement and deposit of immune complex; Dx: concurrent AAV secondary to LAC and associated MN; Tx: prednisone, cyclophosphamide	Levamisole	CEDIA and LC-MS	U	Cocaine metabolite (s) +; cannabinoids, opiates, benzodiazepines, levamisole (c) +	[58]
Hx: 34 y M cocaine user; anamnestic obesity, hyperpigmented lesions, leukocitoclastic vasculitis due to levamisole; recently admitted to ED with proteinuria associated with fatigue/arthralgias Lab: urea 4.6 mmol/L; Cr 71 µmol/L; PCR 449.9mg/mmol; Na+ 140 mmol/L; K+ 4.4 mmol/L; ANA-; ENA-; c-ANCA-; p-ANCA+; HBV/HCV/HIV neg Renal biopsy: no glomerular crescentic or necrotizing injury, glomerular artery wall normal, no artery vasculitis Dx: development of MN after AAV secondary to LAC	Levamisole	CEDIA and LC-MS	U	Cocaine metabolites (s) +;opiates +; benzodiazepines +; levamisole (c) +	[58]
Hx: 68 y M cocaine user, recent haemorrhagic lesions on forearms/face, anamnestic analogous vasculitis 6 months prior Lab: pancytopenia/neutropenia; ANA+; p-ANCA+; anti-PR3+; anti-MPO+ Skin biopsy: leukocytoclastic vasculitis with subepidermal bullae Dx: levamisole-induced vasculitis Tx: 6 months cocaine abstinence	Levamisole *	NA	U	Cocaine +	[47]
Hx: 58 y M polysubstance user presented 4 d after last cocaine use with painful pruritic rash/polyarthralgyas Lab: high CRP, leukopenia; p-ANCA-; c-ANCA-; ANA-; RA-; HCV/HIV neg Skin biopsy:acute/chronic inflammation of superficial derma, acute perivascular inflammation in deeper derma; acute leukocytoclastic vasculitis; eosinophilic infiltrate Dx: cutaneous levamisole induced vasculitis Tx: steroids	Levamisole	LC-MS/MS	U	Cocaine +; levamisole (c)+	[33]
Hx: 40 y F tobacco and cocaine smoker, methadone and hydroxyzine user; anamnestic HCV+, previous septic shock; admitted with extensive retiform purpura/ bullous necrotic lesion legs/nose/ear/cheeks Lab: lactate elevated; PCR 180 mg/L; p-ANCA+; ANA+; LA+; cryoglubulinaemia+; RF+; HBV/HIV neg; HCV pos Skin biopsy: vasculitis, microthrombi/fibrinoid degeneration of vascular walls Dx: transient renal failure, extensive levamisole induced vasculitis	Levamisole	LC-MS/MS	U	Opiates; cocaine; methadone +	[59]
			H segments (*n* = 2)	Levamisole 1–1.78 ng/mg	
Hx: 39 y F intravenous cocaine user dead in bathroom with recent needle marks Autopsy: pulmonary edema, brain/heart/coronary vessels/aorta/kidneys moderately congested COD: cardiorespiratory arrest due to intravenous cocaine and foreign body pulmonary granuloma Histology: foreign bodies (asbestos fibers) in pulmonary parenchyma associated with non-necrotising granulomas in lung Dx: pulmonary granulomatosis due to asbestos fibers related to intravenous cocaine	Asbestos fibers in lung	Histology	VH	Cocaine 40	[53]
			B	BE 300	
Hx: about 20 y M after white drug insufflation (sold as cocaine); presented at ED with hypertension; combative/delirious behaviour; decreased level of consciousness; Clinical: GCS = 7/15–13/15; pin-pupils; systolic pressure = 123 mmHg; HR = 85; Dx: poisonings with LSD after nasal insufflation of a white powder sold as cocaine; Tx: intravenous droperidol; midazolam; intubation (for 12 h).	LSD	UHPLC-Q-ToF	U	Cannabis; benzodiazepine +; ketamine +;	[48]
		UHPLC-MS/MS	B	LSD 60; THC-COOH 38	
Hx: about 20 y M history of cocaine and THC as recreational use; after white drug insufflation (as cocaine); his thoughts were “foggy”; sedation; Clinical: GCS = 13/15; pin-pupils; BP = 130/70 mmHg; HR = 64; vomiting develops hypokalaemia (K 2.9 mmol/L). Dx: poisonings with LSD after nasal insufflation of a white powder sold as cocaine; Tx: anti-emetics; anticonvulsant	LSD	UHPLC-Q-ToF	U	Cannabis +; amphetamines +;	[48]
		UHPLC-MS/MS	S	LSD 60; MDMA 20	
Hx: about 20 y M past recreational use of cocaine/LSD; before white drug insufflation (sold as cocaine); smoked THC; drunk alcohol; reported thoughts-clouding; sensation of dying; vivid hallucinations; sedation; Clinical: HR = 97; BP = 227/145 mmHg; pin-pupils; GCS = 14/15; vomiting developing hypokalaemia (K 2.8 mmol/L); Dx: poisonings with LSD after nasal insufflation of a white powder sold as cocaine; Tx: diazepam; droperidol	LSD	UHPLC-Q-ToF	U	THC +; amphetamines +	[48]
		UHPLC-MS/MS	B	LSD 40; MDMA 10; THC-COOH 22;	
Hx: about 20 y M 10–15 min after white drug insufflation (sold as cocaine) developed hypersalivation; palpitations; nausea; dissociative state; Clinical: pin-pupils; Dx: poisonings with LSD after nasal insufflation of a white powder sold as cocaine Tx: diazepam	LSD	UHPLC-MS/MS	B	LSD < 5; MDMA < 30; THC-COOH < 3	[48]
		
**Heroin**					
Hx: 28 y M 7 yrs cannabis/tobacco use disorders; 2 yrs inhaled heroin use; Due to reduced heroin supply and high cost used “CUT” heroin to save money and discover new drug; ED admission with time/space disorientation, confused; outburst at minimal provocation; pupils constricted/symmetrical; slurring speech, saliva drooling; wide-based gait, staggering/stumbling; unable to perform a tandem walk; Brain MRI: no detectable lesions Lab: ALT 271 U/L; ASL 148 U/L; urea 34 mg/dL; Cr 0.86 mg/dL; anti-HCV-; HCV-RNA 7.27000/mm^3^; HIV/HBV neg Dx: cognitive–behavioural and neurological symptoms due to CUT agent; Tx: sublingual buprenorphine–naloxone for 7 months	Methaqualone, caffeine, oxazepam, Ketazolam, nordazepa, pinazepam, alprazolam, acetaminophen	GC-MS	U	Morphine; benzodiazepines	[55]
			CD	acetaminophen; caffeine; methaqualone; Oxazepam; ketazolam; nordiazepam; pinazepam, alprazolam	
Hx: 28 y M anamnestic with treated bipolar disorder, polysubstance abuse including intravenous heroin, found unresponsive at home with packets of “Santa Muerte”; on arrival typical initial opioid toxidrome Clinical: SpO2 78%; tachycardia, flushing, dry mucous membranes, mydriasis Chest X-ray chest: pneumonia and ARDS CT brain: negative Dx: anticholinergic toxicity after heroin containing 5F-MDMB-PINACA, pneumonia/ARDS Tx: after 2 doses of naloxone, became agitated and combative; OI for 12 d	5F-MDMB-PINACA	LC-MSMS	S	Cocaine +; 6-MAM +; heroin +; fentanyl +; THC +; alprazolam +	[52]
		Immunoassay	U	Cocaine; opiates; fentanyl; THC; benzodiazepines	
		GC-MS and LC-Q-ToF	CD	5F-MDMB-PINACA; Heroin; Fentanyl	
Hx: 25 y M intravenous heroin abuse; admitted at ED for typical opioid toxidrome after intravenous ‘heroin’ injection; Clinical: HR = 102 b/m; BP = 146/89 mm Hg, RR = 24 breaths/min; SpO2 = 98%, flushing, tachycardia, agitation; Dx: anticholinergic toxicity after heroin containing 5F-MDMB-PINACA; Tx: 2 doses of naloxone, became anxious and tachycardic so EV lorazepam was added; intravenous fluids, supportive care. He confirmed Santa Muerte use.	5F-MDMB-PINACA	Immunoassay	U	Opiates+; amphetamine+; barbiturates +; cocaine+	[52]
		GC-MS/LC-Q-ToF-MS	CD	5F-MDMB-PINACA; Heroin; fentanyl	
Hx: 31 y M history of intravenous heroine abuse; admitted at ED after intravenous “heroin” injection associated to typical opioids overdose symptoms; Clinical: HR = 163 b/m; BP = 131/81 mmHg, RR = 29 breaths/minute, SpO2 99%; tachycardic, flushed, dilated pupils; full bladder; Dx: anticholinergic toxicity after heroin containing 5F-MDMB-PINACA, and ARDS; Tx: after naloxone, became combative, anxious, agitated, so intravenous lorazepam was added; intubated for airway protection	5F-MDMB- PINACA	Immunoassay	U	Opiates	[52]
		LC-MS-MS	S	Heroin; 6-MAM, fentanyl	
		GC-MS/LC-Q-ToF-MS	CD	5F-MDMB-PINACA; heroin; fentanyl	
Hx: 25 y M admitted at ED after intravenous heroin (found a drug packet labeled “Santa Muerte”); Clinical: HR = 158 b/m; BP = 215/158 mm Hg; RR = 26 breaths/minute; SpO2 = 99%; urinary retention; anhidrosis; Dx: anticholinergic toxicity after heroin containing 5F-MDMB-PINACA; Tx: naloxone; afterwards tachycardia/agitation; so lorazepam and physostigmine to calm him down;	5F-MDMB- PINACA	Immunoassay	U	Cocaine; opiates; THC	[52]
		LC-MS/MS	S	5F-MDMB-PICA (5F-ADB); heroin; 6-MAM; fentanyl;	
		GC-MS LC-Q-ToF/MS	CD	5F-MDMB-PINACA; heroin; fentanyl	
Hx: 45 y M presented at ED with tachycardia; pinpoint pupils; flushing of skin; drug packet “50 CAL” found in his pocket; Clinical: HR = 124 b/m; BP = 140/82 mmHg; RR = 22 breaths/minute; SpO2 = 99%; pinpoint pupils; flushing of skin; Dx: anticholinergic toxicity after heroin containing 5F-MDMB-PINACA; Tx: midazolam/olanzepine to calm; afterward intravenous diazepam; dexmedetomidine infusion in case of agitation returning; 24 hrs in hospital	5F-MDMB-PINACA	Immunoassay	U	Opiates; fentanyl	[52]
		GC-MS LC-Q-ToF/MS	CD	5F-MDMB-PINACA; heroin; fentanyl	
Hx: 36 y M found unresponsive in the street; after naloxone; agitation; benzo/physostigmine with improvement in agitation behaviour. Clinical: HR = 130 b/M; BP = 160/100 mm Hg; RR = 24 breaths/minute; oxygen saturation 95%; Dx: anticholinergic toxicity after heroin containing 5F-MDMB-PINACA; Tx: naloxone; benzo/physostigmine; intubated to avoid to risk of aspiration from vomiting; found blue packet (“50 CAL”).	5F-MDMB- PINACA	Immunoassay	U	Opiates; fentanyl	[52]
		GC-MS and LC-Q-ToF	CD	Fentanyl; heroin; 5F-MDMB-PINACA	
Hx: 23 y F admitted at ED for severe agitation/combative behavior; Clinical: HR = 156 b/m; BP = 147/64 mm Hg; RR = 20 breaths/minute; Dx: anticholinergic toxicity after heroin containing 5F-MDMB-PINACA; Tx: lorazepam/physostigmine. The patient reported consuming a substance named “50 CAL.”	5F-MDMB- PINACA *	Immunoassay	U	Opiates; fentanyl	[52]
Hx: 27 y M presented at ED after intravenous heroin use; he was admitted with respiratory and CNS depression; Clinical: HR = 130 b/M; BP = 130–94 mm Hg; RR = 22 breaths/minute; sPO2 = 95%; dilated pupils; dry oral mucous membrane Tx: lorazepam; physostigmine. Dx: anticholinergic toxicity after heroin containing 5F-MDMB-PINACA	5F-MDMB- PINACA *	Immunoassay	U	Opiates; fentanyl	[52]
Hx: 36 y F 3 yrs oral opium abuse (dose = 0.3 gr/day); presented at ED with nausea; acute/severe abdominal pain; vomiting; pleuritic chest pain; Clinical: severe hypochromic-microcytic anemia; CT abdomen: multiple radiopaque flakes in intestinal lumen and in its wall due to metal deposition; Dx: lead poisoning in oral opium user; Tx: intravenous chelators (Ca Na_2_ EDTA)	lead	NA	B	Lead 780; lead (7 day after) 460	[49]
**Γ-hydroxybutyrate (GHB)**					
Hx: 29 y M and 34 y M bought two GHB doses that were dissolved in 200 mL of alcohol each; 1st case drunk the whole drink in 10 min; 2nd case drunk 2/3 of his drink; Clinical: after 20 min 29 y began to accuse chest pain; tachycardia; short of breath; 34 y accused just headache. Dx: acute intoxication GHB and SLD	SDF		B	GHB 55–100 (case 1); 37–800 ng/mL (case 2); SDF 340 (case 1), SDF< LOQ; desMe-SDF < LOQ; desMe-SDF < LOQ	[50]
			U	GHB: 35;700 and 15;500; SDF = 1;270 and SDF 1;220 desMe-SDF = 810 and 1;210	
			Drink	GHB 7460;7 μg/mL; SDF 521;2 μg/mL	
**Alprazolam**					
Hx: 49 y M past substance abuse; found dead in his bed; nearby the corpse; a glass with dried; white; crystalline substance; on a shelf/living room; 2 plastic-bags containing white tablets (imprint XANAX); Autopsy: heart; lungs; liver; kidney: sign of decomposition; COD: acute intoxication of caffeine and etizolam.	Etizolam and caffeine	GC-MS and HPLC-DAD	FB	Etizolam = 770; caffeine = 190 Etoh 24 mg/mL	[51]
			CB	Caffeine 426 mg/mL	
			U	THC-COOH 192	
			H segments (*n* = 4)	Etizolam 0.05–0.11; THC 0.06–0.19; amphetamine 0.42–2;568; cocaine 0.03–0.22. BEG 0.04–0.07	
			Stomach content	Etizolam +	
			Drug	Caffeine; etizolam	
**Kratom**					
Hx: 54 y M past history of HCV treated; alcohol use disorder; OUD; admitted at ED with altered mental status after 1 spoon of “Kratom crazy^®®^” taken for an entire year; in morning he added a table spoon of “Vivazen Botanical De Kratom^®®^”; headache; vomit; fall asleep; when he woke up; he had incomprehensible speech. Clinical: BP = 120/70 mmHg; HR = 70; RR 18; sPo2 = 98%; Lab: glucose = 124 mg/dL; leukocytosis; troponin T = 91 ng/L; CT brain: large right frontal intraparenchymal haemorrhage with extension to ventricles (40 cm^3^) and mass effect; MRI brain: intraparenchymal hemmorrhage with 7 mm midline shift. Dx: PEA-adulterated kratom intoxicationTx: surgical craniotomy	PEA	LC-MS/MS	S	Mitragynine 340	[56]
		LC-HRMS/MS	CD	PEA	

+, positive at screening test; *, suspected without toxicological confirmation. Abbreviations: AAV, ANCA-associated vasculitis; AKI, acute kidney injury; c-ANCA, cytoplasmatic-anti neutrophil cytoplasmic antibody; BE, benzoylecgonine; B, blood; CB, cardiac blood; CD, confiscated drug; Cr, creatinine; CSF, cerebrospinal fluid; CIMDLs, cocaine-induced midline destructive lesions; COD, cause of death; CRP, c-reactive protein; Dx, diagnosis; EME, ecgonine methyl ester; ESR, erythrocyte sedimentation rate; FB, femoral blood; H, hair; HR, heart rate; Hx, history; LA, lupus anticoagulant; LAC, levamisole-adulterated cocaine; MN, membranous nephropathy; NA, not available; OI, orotracheal intubation; p-ANCA, perinuclear-anti neutrophil cytoplasmic antibody; PEA, phenylethylamine; RF, rheumatoid factor; RFA, retinal fluoroangiography; RR, respiratory rate; SDF, sildenafil; SpO2, oxygen saturation; S, serum; SS, Susac syndrome; THC-COOH, 11-Nor-9-carboxy-Δ9-tetrahydrocannabino; Tx, treatment; U, urine; VH, vitreous humour; WCC, white cell count; (s), screening; (c), confirmation.

**Table 3:** Number of authentic cases of adulterated cocaine, heroin, and new psychoactive substances (NPS) intoxications reported in the literature between 2017 and 2022.

Number of Cases	Adulterant	Analytical Technique	Matrices	LOD (ng/mL)	LOQ (ng/mL)	Quantification (ng/mL)	Reference
DUID (*n* = 724)	Levamisole; PTHIT	LC-MS/MS	H	2.5 pg/mg	10 pg/mg	Cocaine 500–800.000 pg/mg (*n* = 627); PTHIT 3.5–61,000 pg/mg; levamisole/dexamisole 0.71–1.34	[43]
DUID (*n* = 55)	Caffeine DZ; hydroxyzine; levamisole; lidocaine; benzocaine; diphenhydramine; phenazone; procaine	SWATH LC-HRMS	U	__	__	THC + (*n* = 19) THC, cocaine + (*n* = 19); cocaine + (*n* = 17)	[65]
U (*n* = 100) PL (*n* = 8)	Levamisole Aminorex	LC-HRMS	U PL	___	1 0.1	U: levamisole (*n* = 72) average conc.= 565 (4–72,970 µg/L); BE (*n* = 100) averange conc. = 13,510 (174–251,000 µg/mL); Aminorex not detected U: metabolites: 4-OH-LEV; LEV sulfoxide; LEV glucuronide; OH-LEV glucuronide; PL (*n* = 8): LEV average conc. = 10.6 (0.9–64.1 µg/L); U (*n* = 8) average conc. =144.50 (50–10,050); aminorex not detected	[66]
H (*n*= 100) CRATOD admission	THC; lidocaine; phenacetin; levamisole; benzocaine; procaine hydroxyzine	LC-MS/MS	H	___	___	Cocaine (*n* = 100), BE + (*n* = 100); CE + (*n* = 94) THC + (*n* = 23); lidocaine (*n* = 92) +; phenacetin + (*n* = 69); levamisole + (*n* = 31) benzocaine + (*n* = 19); procaine + (*n* = 5); hydroxyzine (*n* = 2)	[67]
Brain tissue (*n* = 10) Blood (*n* = 5) U (*n* = 1)	DZ; hydroxyzine; levamisole; lidocaine; phenacetin; procaine; cetirizine	GC-MS	Brain tissue; U; BL	Cocaine 3.6 BE 27 EME 67 CET 71 DZ 26 HYD 9 LEV 14 LID 38 PHE 21 PRO 16	Cocaine 30 BE 93 EME 105 CET 363 DZ 101 HYD 152 LEV 66 LID 81 PHE 132 PRO 121	Cocaine 530.8 ng/g; benzodiazepine 423.1 ng/g; EME 548.7 ng/g; LEV 128.1 ng/mg; LID 73.5 ng/mg; HYD 170.5 ng/g; ALC (BL) 0.86‰; ALC (brain) 0;54‰; ALC (U) 4;97‰	[65]
U (*n* = 3665)	Levamisole	Immunoassay LC-Q-ToF	U	___	___	Cocaine + (*n* = 51); levamisole + (*n* = 27); methamphetamine/MDMA + (*n* = 23); cannabis metabolites + (*n* = 12); heroin or metabolite + (*n* = 2).	[69]
H (*n* = 55)	Levamisole	LC-MS/MS	H	___	Levamisole 0.002 pg/mg; Cocaine 0.005 pg/mg	H [cocaine + samples > 500 pg/mg]: 4265.4 pg/mg (levamisole); 322.9 pg/mg (MDMA); 253.9 g/week (alcohol); H [cocaine—samples]: 0 (levamisole); 0.99 pg/mg (MDMA); 56.5 g/week (alcohol)	[68]
**Heroin**							
H (*n* = 40)	Fentanyl; acetylfentanyl; furanylfentanyl; U-47700	UHPLC-MS/MS	H	0.1–0.3 pg/mg	0.3–0.9 pg/mg	Fentanyl 2.3–8600 pg/mg (mean = 860 pg/mg; median = 440 pg/mg); acetylfentanyl = 2.1–3200 pg/mg (mean = 160 pg/mg; median = 26 pg/mg); furanylfentanyl = 0.7–42 pg/mg (mean = 8.0 pg/mg; median = 1.6 pg/mg); U-47700 (*n* = 3/40): 1.4–4.5 pg/mg	[70]
OF (*n* = 30) U (*n* = 30)	Fentanyl; norfentanyl; acetylfentanyl; U-47700	LC-Q-ToF-MS	OF U	Fentanyl 1; norfentanyl 2; acetylfentanyl 1; carfentanil 1; U-47700 1	___	U (*n* = 29 fentanyl +); OF (*n* = 27 fentanyl +); norfentanyl; acetylfentanyl; U-47700	[71]

Abbreviations: COC= cocaine; BL = blood; BE= benzoylecgonine; CET = HYD metabolite cetirizine; CRATOD, crack cocaine addiction treatment; DZ = diltiazem; EME = ecgonine methyl ester; H = hair; HYD = hydroxyzine; LC-Q-ToF-MS = liquid chromatography quadrupole time-of-flight mass spectrometry; OF = oral fluid; PL = plasma; PTS = patients; phenyltetrahydroimidazothiazole, PTHIT; U, urine.

## 3. Discussion

Adulteration exposes drug consumers to unexpected threats, due not only to unintentional exposure to a potentially harmful substance but also due to pharmacological interactions between drugs and adulterants [75,76].

Similar to fluctuations in the availability of drugs of abuse, drug adulteration trends also may fluctuate in response to economic and political factors influencing the availability of certain substances [4].

The reported data disclosed new adulteration practices, although the seized samples analyses revealed the presence of well-established adulterants such as levamisole for cocaine or paracetamol/acetaminophen for heroin [5]. In 2022, quinine and quinidine were of particular concern in the USA, due to detection in more than 2,000 street drug samples. However, the reported data showed that quinine and quinidine were already used as cocaine adulterants in 2017 and 2018 [76]. Fentanyl was commonly detected as an adulterant in heroin samples in the USA and in Canada, presumably to enhance drug potency and reduce the amount of heroin needed for each dose [12,38,42,73].

NPS adulteration occurs not only with all classic drugs of abuse but also with other NPS. The wide use of these compounds as adulterants may be related to the ease of distribution due to uncontrolled status and ease of production since they are produced in “kitchen laboratories”. However, it is not clear if certain NPS may be byproducts of the manufacturing process, such as fentanyl in butyrlfentanyl samples seized in Sweden between 2010–2014. Seized samples of kratom, a psychedelic plant containing mitragynine and 7-OH-mitragynine, were adulterated with caffeine and synthetic O-desmethyltramadol (ODT) and sold under the name “Krypton” [77]. ODT, a bioactive metabolite of tramadol, was added to mimic the sedative-narcotic effects of kratom [78,79]. Other adulterated kratom products contained high concentrations of 7-OH-mitragynine. Usually, 7-OH-mitragynine is less than 2% of the mitragynine concentration in kratom; however, in this case, 7-OH-mitragynine was added as an adulterant to increase opioid-like effects [80]. Other synergistic effects were observed in the case of synthetic cannabinoids added to THC [34]. The addition of fentanyl to methamphetamine may lead to greater dependence on the mixture. 

Although the reported data covered a limited time range, more recent evidence confirms the changing nature of adulteration practices, such as the xylazine-laced heroin seized in the USA [81,82].

Adulterants may play a role in the adverse effects commonly related to the primary drug, such as vasculitis induced by levamisole-adulterated cocaine [83]. P/c-ANCA positivity suggests that levamisole-induced vasculitis has an autoimmune mechanism. Immune pathologies require glucocorticoids, as was the case of a 29-year-old suffering from multifocal white matter lesions with brainstem and cerebellar involvement after cocaine/levamisole consumption, requiring immediate treatment with corticosteroids [84]. Noteworthy, p/c ANCA positivity was used as evidence of levamisole exposure in the presence of analytically confirmed cocaine positivity. Although, the unexpected negativity of p-c/ANCA despite vasculitis [5] in a case of certain cocaine consumption suggests that toxicological levamisole confirmation in biological matrices is necessary to prove consumption. The most challenging aspect of levamisole quantification in biological matrices may be its short half-life (5–6 h). In these cases, hair analysis may offer the best approach to document exposure to short half-life substances [45,59,67,68,85]. Interestingly, levamisole and cocaine quantification in post-mortem brains suggests they may have synergistic negative effects on the encephalic white matter [68]. Moreover, this synergistic effect was reported in living patients by diffusion tensor imaging and magnetic resonance imaging [86,87] revealing that levamisole increases the risk of white matter damage [87]. 

Although other adulterants were detected in seized samples, such as phenacetin, benzocaine and lidocaine, these substances were not reported in clinical cases, probably due to the lack of specific analyses of compounds other than principal drugs causing health threats. Noteworthy is the alarming increase in cocaine-related deaths due to fentanyl-adulterated cocaine [88]. Huhn et al. reported the case of a man who continued to test positive for fentanyl in urine for up 19 days [89]. Surprisingly, the new trend of heroin adulteration with synthetic cannabinoids and/or fentalogues was reported in a number of cases. In fact, 5F-MDMB-PINACA and fentanyl were reported in a case series in which the adulterants were confirmed by urine analysis [52]

Apparently, lead adulteration of drugs such as opium, heroin and methamphetamine is a common practice in Iran [49]. In lead-adulterated drugs cases, the diagnosis is always difficult because of unspecific abdominal symptoms caused by chronic lead intoxication. The diagnosis of lead poisoning requires a clear clinical suspicion and sophisticated technological methods to detect it. It is important to highlight lead adulteration of opium because of its potential spread to Europe.

Rarely, cases of LSD-adulterated methamphetamine were reported, characterized by the inconsistency between the type of symptoms appearing in the acute intoxication phase and the substance reportedly taken by the patient. This inconsistency led to toxicological research to detect LSD and adulterants [48]. The state of well-being induced by MDMA including increased activation and emotional excitation is associated with a better response to LSD. The combination of MDMA/LSD enhances the psychedelic experience by inducing positive emotions. On the other hand, this has a minor enhancing effect relating to psychedelic experience. Club drugs, such as methamphetamine, MDMA, ketamine, cocaine, and GHB are often taken in combination with sildenafil, especially when sexual encounters are anticipated [50].

## 4. Method

A systematic literature search was performed in PubMed and Scopus databases and official international organizations’ websites; according to PRISMA guidelines (12). Keywords “drug of abuse”; “new psychoactive substances”; “fentanyl analogues”; “heroin”; “cocaine”; “amphetamine”; “THC”; “cannabis”; “stimulants”; “synthetic cathinones”; “synthetic opioids”; “opioids”; “opiates”; “phenethylamine”; “synthetic cannabinoids”; were combined with “adulteration”; “adulterants”; and “contaminated”. A total of 3604 scientific articles (1250 from PubMed and 2354 from Scopus) published from 2017 to 2021 were initially screened for eligibility. Two scientists individually evaluated each entry from one database; considering for full text reading only titles and abstracts mentioning analytical assessment of drugs and adulterants in seized materials and/or in drug-related intoxications. Further screening excluded studies according to the following criteria:

(1) Articles not written in English
(2) Commentary; editorial letters; and surveys
(3) Duplicates were removed
(4) Irrelevant studies

Articles were classified by matrix investigated as “biological specimens” or “non biological specimens”. Finally, articles were excluded for insufficient data.

## 5. Conclusions

Drug of abuse adulteration is a common practice involving a wide range of different active substances added to the primary drugs for pharmacological or profit purposes. The data showed that adulterants are widely added to all the classes of drugs of abuse, including NPS. Furthermore, NPS may be used as adulterants to potentiate the primary drug effect or obtain cocktails exerting unexpected effects on the abusers. However, they represent an additional threat to the users’ health since a synergistic effect is observed in the comparison of certain intoxication symptoms. Moreover, the unexpected presence of certain psychotropic drugs may lead to incorrect treatment of intoxication cases. For these reasons, adulteration practices must be monitored to assess changes in the drug supply and adulterants should be analytically assessed in intoxication cases.
